# Age, exercise, and the outcome of sepsis

**DOI:** 10.1186/s13054-017-1840-9

**Published:** 2017-11-23

**Authors:** Debasree Banerjee, Steven M. Opal

**Affiliations:** 1 Department of Medicine, Warren Alpert Medical School of Brown University, 593 Eddy St., Providence, RI 02903 USA; 2 Lifespan Hospital System, Providence, RI USA

**Keywords:** Sepsis, Septic shock, Aging and immunity, Immunosenescence, Exercise and infections, Sepsis in the elderly

## Abstract

We report on the increasingly important need to diagnose and care for the elderly with sepsis as a distinct patient population. We share an overview of age-related changes in sepsis physiology and the potential role of exercise.

See related research by Tyml et al., https://ccforum.biomedcentral.com/articles/10.1186/s13054-017-1783-1

For all the supposed benefits attributable to moderate exercise in the elderly population (better cardiovascular fitness, improved microcirculation, less obesity, etc.) [1–3], improved outcome from sepsis has not been listed among them until now [4]. At least in inbred, old mice, voluntary exercise appears to provide a survival benefit from experimentally induced intra-abdominal sepsis. If these results could be translated into aging human populations, the ramifications would be substantial indeed.

Sepsis is a critical issue in old age and is among the top causes for intensive care unit (ICU) admission in the elderly [5]. The incidence of sepsis in our aging population is predicted to rise precipitously in the next decade as the “baby boomer” population reaches old age. Older adults and particularly nursing home residents have higher rates of ICU admission, longer length of stay, and increased hospital mortality than younger adults [6].

The mechanism(s) underpinning the disproportionate susceptibility to sepsis in the elderly is not yet fully understood. Elderly patients are at higher risk for sepsis likely related to the higher rates of comorbidities, including dementia, poor dentition, and diabetes. With age, intact integument and physiologic reflexes (intact cough reflex, balance, and mobility) that contribute to the body’s physical defense mechanisms to infection can degrade. Additionally, institutionalization, implanted devices, and surgical procedures can predispose patients to infection [7].

All mammals age over their lifetime, manifest by gradual development of telomere shortening of chromosomes, degenerative arthritis, loss of hair, inactivity, atherosclerosis, impaired T- and B-cell responses, and age-related malignancies [7, 8]. Mice age over a 2-year lifespan rather than the typical 80-year lifespan of humans, making mice a convenient animal model to study the effects of aging on a range of pathophysiologic responses.

Elderly septic patients often do not have typical clinical responses to sepsis, making diagnosis by the Sepsis-3 quick Sepsis-Related Organ Failure Assessment (qSOFA) criteria difficult. Older patients may present with inverse signs and symptoms (hypothermia, leukopenia), or nonspecific signs of infection [6]. Accepted thresholds for biomarkers levels for diagnosis may not be appropriate in the older population, particularly among patients with pre-existing organ dysfunction. Thus, given the protean manifestations of infection in the elderly, diagnosis of sepsis is often delayed.

Once infected, older patients experience a disadvantaged host immune response with defects in humoral and cellular immune function [9]. The pattern of cytokine release during sepsis in the elderly demonstrates higher levels of tumor necrosis factor (TNF)-α, interleukin (IL)-1β, and IL-6 [7, 10]. A smoldering inflammatory state during aging termed “inflammaging” can affect sepsis physiology [11]. However, despite evidence of increased baseline inflammation, after insult, an immunosuppressed state is commonly observed in the elderly. This is part of the “immunosenescence” of aging, which results in quantitative and qualitative changes in lymphocytes (CD4, CD8 T cells and B cells) and myeloid cells [12, 13]. Specifically, elderly patients experience attenuated cell-mediated immunity in the form of a truncated T-cell repertoire, decreased IL-12 release, decreased lymphocyte proliferation, and dampened signal transduction. Humoral immunity is also adversely affected with aging. Decreased B-cell function with blunted antibody responses to neoantigens is commonplace due to lack of helper T-cell signals and decreased expression of costimulatory molecules [13].

Elderly mice experience many of the same age-related defects in immune function and increased risk of infection as observed in the older adult human patients [7]. Aged mice are more susceptible than young mice to endotoxin-induced systemic inflammation, and are more likely to succumb as a result of systemic infection in pneumonia models and from intra-abdominal infection following cecal ligation and puncture [8]. While it might be speculated that exercised old mice in good cardiovascular fitness might fare better in sepsis than nonexercised, aged mice, limited experimental data exist to answer this question.

A recent report now directly challenges the potential role of exercise in an isolated, controlled setting where other variables were minimized [4]. These investigators induced sepsis by an intraperitoneal injection of a fecal slurry in male, nonobese C57BL/6 mice. Exercised older mice (22 months old) had a better outcome with significantly lower systemic cytokine and chemokine response and had a less pronounced procoagulant response than nonexercised, aged mice subjected to the same infectious challenge.

Intriguingly, consistent upregulation of endogenous endothelial nitric oxide synthase (eNOS) was observed in exercised mice compared to nonexercised mice [4]. Nitric oxide is a potent vasodilator and has the capacity to limit neutrophil and platelet adherence to endothelial surfaces [14]. Exercise in older human subjects is known to improve endothelial function, increase eNOS expression, and promote microcirculatory responsiveness [15].

Like many innovative investigations, the results of this study pose more questions than they answer. Would the advantageous effects of exercise work as well in outbred mice, females (both premenopausal and postmenopausal), and how long does the protective effect of exercise last in aged mice after exercise succession? How much exercise is enough, when should it start, and is there a dose-response effect to exercise conditioning? Does exercise work better in obese or nonobese subjects? The most critical question is, of course, how well do these study findings in mice translate to improved outcomes in human sepsis? The data collected thus far in older humans indicate that while the effects of exercise on inflammatory biomarkers is measurable, the effect size is rather small and often confounded by obesity indices [1–3].

Should future surviving sepsis campaigns recommend daily exercise for older patients to stave off the risk of death from sepsis potentially lurking in their future? Should octogenarians be encouraged to do some daily exercise? Based on this study, one of the purported benefits might be a more constrained and appropriate host response to systemic infection.

Additional animal models would likely be needed to confirm these findings as retrospective, observational studies in humans would be susceptible to selection bias and randomized trials would be very difficult to organize. Randomly assigning people to the nonexercise group would pose an ethical challenge considering the many common benefits of light physical activity in aging populations [3]. If confirmed in further studies, we might find ourselves someday encouraging our senior citizens to prevent sepsis by giving up their rocking chairs for the exercise bike and the jogging trails.

## Abbreviations

C57BL/6: Black 6 mouse
CD: Cluster of differentiation
eNOS: Endothelial nitric oxide synthase
ICU: Intensive care unit
IL: Interleukin
qSOFA: Quick Sepsis-Related Organ Failure Assessment
TNF: Tumor necrosis factor
